# Does Attentional Focus Influence Psychophysiological Responses to an Acute Bout of Exercise? Evidence From an Experimental Study Using a Repeated-Measures Design

**DOI:** 10.3389/fphys.2021.680149

**Published:** 2021-06-25

**Authors:** Friedrich Meixner, Cornelia Herbert

**Affiliations:** Applied Emotion and Motivation Psychology, Institute of Psychology and Education, Ulm University, Ulm, Germany

**Keywords:** aerobic exercise, cycling exercise, attentional focus, psychophysiology, impedance cardiography

## Abstract

Attentional focus during aerobic exercise has been studied in the context of sports performance, injury prevention and affective experience. Previous research suggests that an additional mental task parallel to the physical activity might influence exercise experience and performance. It has been tested if attentional focus influences cardiovascular activity, positive/negative affect, and subjective exertion during a cycling exercise. Data from *N* = 30 female participants has been collected using a repeated measures design, with the following experimental manipulations: (A) an internal attention focus (i.e., paying attention to force production of the quadriceps muscles), (B) an external attention focus (i.e., paying attention to changes in brightness in the cycling track simulation), and as control conditions, (C) exercise without attention focus (i.e., no specific instruction was given) and (D) no exercise, no attention focus. Subjective affect and subjective exertion were assessed, and changes in cardiovascular activity were recorded via mobile impedance cardiography (ICG) at rest, during and after the exercise, including HR, HRV (RMSSD, HF), PEP, CO, SV, LVET, and RSA. Exercise was associated with adaptations in cardiovascular activity, positive/negative affect, and subjective exertion. However, this did not interact with attentional focus. The original hypothesis could not be supported: instructed attentional focus does not influence affect, exertion, or cardiovascular activity during a cycling exercise. Therefore, attentional focusing during exercise does not appear to put notable additional mental demands on the physically active participant. Nonetheless, impedance cardiography delivered reliable measurements even during the cycling exercise.

## Introduction

Acute exercise can lead to an increase in positive affect and relief of negative symptoms, e.g., depressive symptomatology (Frühauf et al., 2016), fear or anxiety (Bibeau et al., 2010). However, acute exercise can also lead to negative subjective experiences, i.e., fatigue, or feelings of excessive exertion. Being crucial to the future adherence to exercise regimens (Hall et al., 2002; Ekkekakis et al., 2008, 2011; Zenko et al., 2016; Decker and Ekkekakis, 2017), it is important to identify factors that are reducing or unnecessarily increasing negative feelings during exercise.

Attentional focus has been suggested as a potential influence on affect and subjective exertion during aerobic exercise (Lind et al., 2009; Brick et al., 2014). Current theoretical frameworks classify two dimensions of attention during exercise: internal/external and task-relevant/-irrelevant (Stevinson and Biddle, 1998, 1999; Brick et al., 2014). Exercising at high intensities, i.e., beyond the ventilatory threshold has been shown to be aversive (Ekkekakis and Petruzzello, 1999; Ekkekakis et al., 2004), usually enforcing an internal focus of attention (e.g., Hutchinson and Tenenbaum, 2007). At moderate intensities, attentional focus seems to be controllable and possibly modulating affective experience (Lind et al., 2009; Brick et al., 2014). Although the relationship between attention, intensity and affect is described by numerous studies, their association with physiological changes during exercise remains largely undiscovered.

Cardiovascular changes elicited during aerobic exercise can be summarized as (A) an increase in heart rate (HR), (B) a decrease in heart rate variability (HRV; especially HF/RMSSD/RSA as signs of vagal/parasympathetic withdrawal; see Billman, 2013; Heathers, 2014), (C) an increase in cardiac output (CO) and stroke volume (SV), and D) a shortening of the systolic time intervals (STI) pre-ejection period (PEP) and left-ventricular ejection time (LVET), compared to baseline values at rest. Cardiovascular recovery can be broadly described as the reverse effects of A-D), i.e., each parameter returning to baseline levels after the exercise (for a review on cardiovascular regulation during exercise, see Michael et al., 2017). All of these parameters can be non-invasively measured via impedance cardiography (ICG; Willemsen et al., 1996).

Besides adaptation to meet physical demands, cardiovascular activity is also influenced by psychological factors such as cognitive demand (Montoya et al., 1997), emotions (Kreibig, 2010), stress (Steptoe et al., 1993; Al’Absi et al., 1997), effortful coping (Kelsey, 2012), or affect (Papousek et al., 2010; Radstaak et al., 2011). Psychological influences on cardiovascular activity can run counter or in the same direction as the effects of exercise, e.g., during a demanding task such as mental arithmetic, sympathetic activity as indicated by STIs such as PEP, rises to meet increased demands (Montoya et al., 1997), but during psychologically stressful tasks, such as the cold-pressor test, CO is decreased and vascular resistance is increased (Montoya et al., 1997). However, psychophysiological reactions as an effect of attentional focus while exercising have previously not been examined comprehensively.

Combining physical activity with a specific attentional focus might place additional cognitive demands on the active person, possibly eliciting a greater sympathetic reaction to supply necessary resources, compared to exercise without an instructed attentional focus (e.g., Roth et al., 1990; Taelman et al., 2011). However, this has not been consistently reported: It has been suggested that there are no additive effects of combined mental and physical challenges on psychophysiological reactions (Wasmund et al., 2002), but also that physiological responses to physical activity might be blunted or masked by a parallel mental task due to attention being directed away from the physical task (Greig et al., 2007).

Attentional focus is a widely used experimental manipulation (or analyzed correlate) in sports psychology. However, previous literature seldom acknowledged the possibility that attentional focus itself could be considered an additional task, adding mental demands on top of the physical demands of an exercise. This might be even more true for non-professional athletes, which are less experienced with the employment of an attentional focus. Our study was conducted to investigate a possible additive mental demand to an exercise by instructing a specific attentional focus.

We examined the impact of attentional focus on subjective exertion, affect and on physiological changes due to acute aerobic exercise in female non-professional exercisers, i.e., healthy, active adults not engaged in regular performance training or athletic competitions. Sample selection is critical due to possible gender-dependent responses to attentional focus (Frazier and Fatis, 1980; Wrisberg et al., 1988; Lind et al., 2009), differing reasons for exercise (e.g., see Furnham et al., 2002), physiological reactions to exercise (Roepstorff et al., 2002; Acevedo, 2012; Hill et al., 2018), as well as interactions with the experimenter’s gender possibly influencing exertion reports for psychosocial reasons (e.g., for interactions on experimenter’s gender and reported pain, see Levine and De Simone, 1991; Aslaksen et al., 2007).

Previous literature has not been uniformly manipulating attention experimentally, but also assessing self-reported attentional focus. Since theoretical categories of internal and external focus of attention are conceptually broad, it is crucial to experimentally manipulate an internal and an external focus of attention during exercise precisely, to maintain comparability between participants. Furthermore, the effects of an instructed attentional focus might differ from the effects of a voluntarily chosen attentional focus, possibly even if it focuses on the same stimulus category, since the former requires adhering to a specific additional task. It is therefore sensible to assess self-reported focus of attention in a separate condition without any attentional focus instructions.

We were particularly interested in replicating previous findings regarding the effects of an internal vs. an external focus of attention on subjective affect and subjective exertion using an experimental design, while also investigating whether an instructed, consistent focus of attention can be considered beneficial in terms of affect and subjective exertion compared to exercising with a voluntarily chosen, flexible focus of attention. Secondly, psychophysiological effects of the additional task of attentional focusing should be assessed, leading to the following open questions:

1a. In non-professional exercisers, instructing an internal focus of attention should lead to increased subjective exertion and reduced positive affect during and after exercise, compared to exercise with an instructed external attentional focus.

1b. Exercise without any attentional focus instruction should lead to less subjective exertion and higher positive affect compared to both exercise conditions with an instructed focus of attention, due to the latter’s combined mental and physical demands.

2. Since previous research has mainly employed mental arithmetic as an additional mental task, and results were discordant, the following hypothesis is explorative: Instructing an attentional focus should lead to additional mental demands, compared to aerobic exercise without attentional focus instructions. This should be visible in an increased sympathetic activity during exercise, and slower parasympathetic reactivation during recovery due to combined demands, compared to the exercise control condition. To assume a systemic point of view, all physiological parameters assessable via ICG are considered: An increased sympathetic reaction should be visible in a decreased PEP, and an increased CO and SV. However, CO and SV are expected to be counter-regulated by an increased vascular resistance as a reaction to the additional mental demands placed on the participant by instructing an attentional focus. Therefore, a decreased PEP and a blunted SV/CO response would be indicative of additional mental stress during the exercise.

## Materials and Methods

### Participants

A required sample of *N* = 26 has been calculated to identify medium effects using G^∗^Power 3.1 (Faul et al., 2009): *f* = 0.25, α = 0.05, 1-β = 0.95, 1 group, 4 measurements ANOVA, ε = 0.8.

*N* = 32 female participants were recruited via advertisements around university campus or mailing lists. Inclusion criteria were being 18–30 years of age, right-handed, physically able to exercise, non-smokers, native German speakers and not regularly exercising for more than 5 h per week (i.e., greatly exceeding the WHO recommendations for a healthy lifestyle), nor preparing for any sports-related competition. Menstrual cycle and use of contraceptives have been assessed, but did not lead to participants’ exclusion (recent meta-analyses suggest that influence of menstrual cycle (McNulty et al., 2020), as well as oral contraceptives (Elliott-Sale et al., 2020) on exercise performance in women is trivial and not sufficient to form general guidelines). Two participants dropped out of the experiment after two sessions due to skin irritation caused by the cycling exercise. Participants received course credit for their participation.

### Experimental Design and Procedure

The experiment was devised as a repeated-measures design. Each participant gave written informed consent and filled in an online questionnaire. On four different days, across 4 weeks, participants then took part in four different experimental sessions in the laboratory, in randomized order. In three conditions, they completed a 22 min cycling exercise (5 min warm-up, 15 min exercise, 2 min cool-down) on a stationary bicycle ergometer (Daum, ergo_bike premium8i) in front of a simulated, plain track alongside the Rhine river, projected with a diameter of ∼2.5 m. The simulation was displayed using the ErgoPlanet software^1^. Simulation speed was adaptive to participants’ cycling speed. The track simulation contained the Rhine river, trees, other cyclists, and different lightings to provide a natural exercise setting and external stimuli for participants to focus on.

**FIGURE 1:**
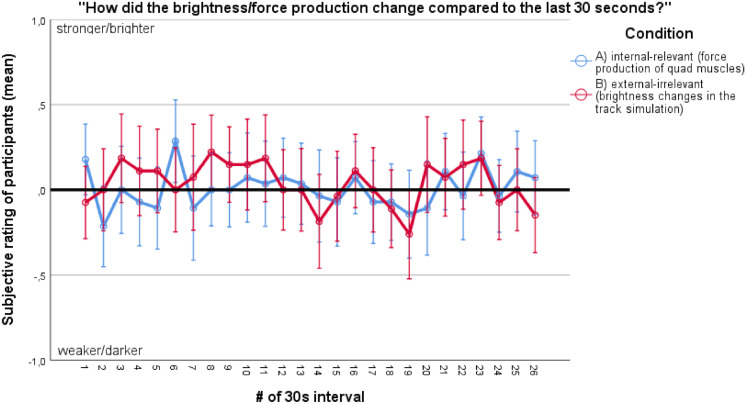
Participants’ mean subjective rating on change in the intensity of internal-relevant or external-irrelevant stimuli during the course of the cycling task. Vertical bars denote +/− standard errors.

In each of the experimental conditions, participants received different instructions (Schücker et al., 2009, 2016a,b). During the internal focus condition A, participants were instructed to focus on the force production of their quad muscles. During the external focus condition B, participants were instructed to focus on brightness changes in the track simulation (Schücker et al., 2016a). Participants received pre-recorded auditory instructions every 30 s on where to direct their attentional focus and had to state out loud if they felt that force production (A) or brightness (B) has changed in the last 30 s. Participants’ answers were controlled by the experimenter (see Figure 1). During the exercise control condition C, participants were simply instructed to cycle for 15 min and received no auditory instructions/prompts afterward. In condition D (non-exercise control condition), participants were instructed to sit passively on the ergometer, and to refrain from any cycling movements.

Experimental sessions lasted ∼90 min. All sessions were done over the course of 4 weeks, at the same time of day for every participant. All sessions were performed under the supervision of female experimenters. An overview over the design is given in Table 1.

**TABLE 1:**
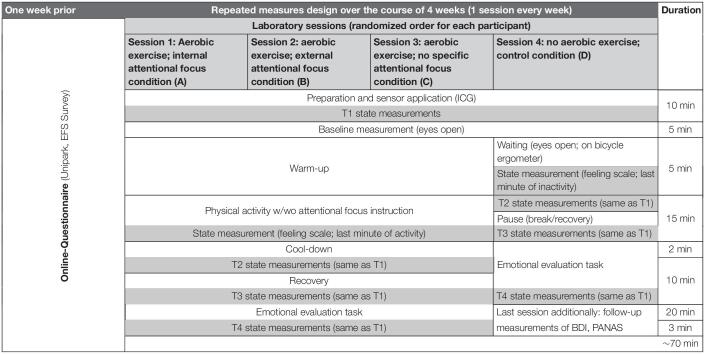
Experimental design; order of laboratory sessions has been randomized for every participant.

#### Online Questionnaire

In the online questionnaire (presented using the platform EFS Survey, Unipark) preceding participation, participants answered anamnestic and demographic questions. In addition they filled in the Physical Activity Readiness Questionnaire (PAR-Q; Thomas et al., 1992). If participants met the inclusion criteria, they were invited to fill out self-report questionnaires (see Materials). Afterward, laboratory appointments were scheduled, and reminders were sent every week.

#### Laboratory Sessions

**FIGURE 2:**
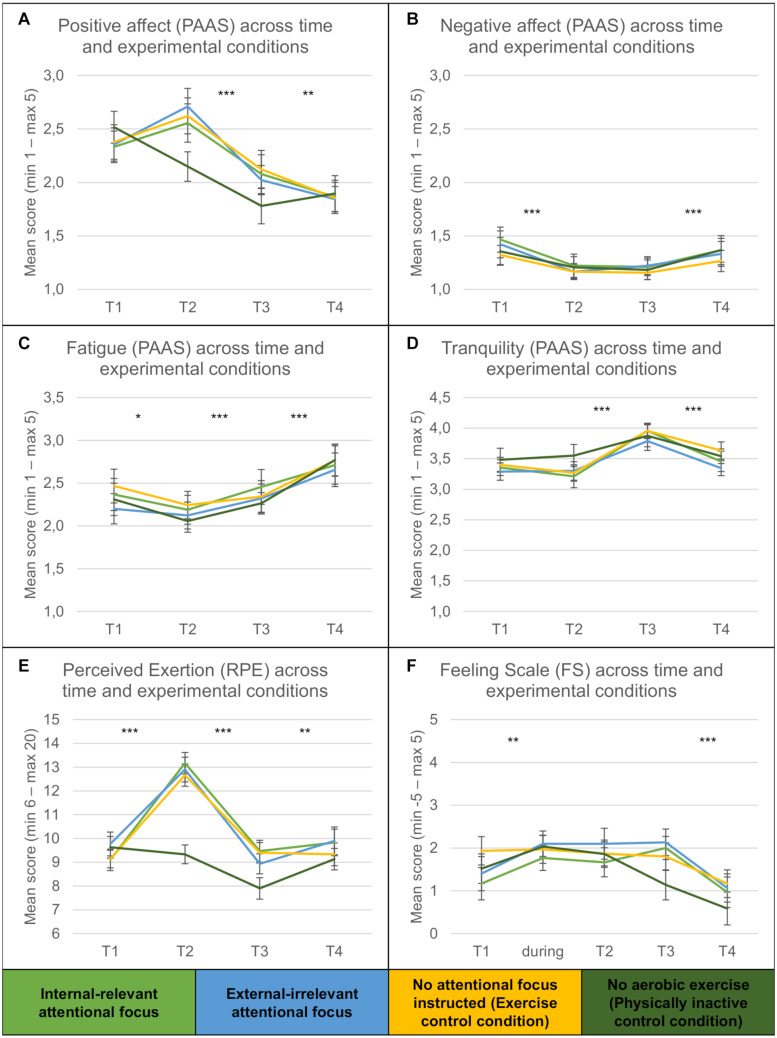
**(A–F)** Positive affect (range 1–5), negative affect (1–5), fatigue (1–5), tranquility (1–5), rating of perceived exertion (6–20), and subjective positive feeling (−5 to 5) before (T1), after (T2), + 10min after (T3), + 30min after (T4) the cycling exercise. Asterisks indicate significant differences between time points, regardless of exercise condition (*p < 0.05; **p < 0.01; ***p < 0.001). Condition D (physically inactive) is depicted for additional reference. Vertical bars denote +/− standard errors.

Each of the four laboratory sessions had the same structure. Upon arrival, participants were asked if they felt fit to be physically active. Afterward, electrodes were attached to measure impedance cardiography (ICG) and a 5-min baseline measurement (seated on a chair) took place. In the exercise conditions ABC, a 5-min warm-up phase on the ergometer followed, during which participants cycled at ∼60 RPM, while the investigator raised the resistance (Watt) until moderate intensity, i.e., the individually targeted heart rate has been reached^2^ (60–70% of max heart rate according to Tanaka et al., 2001). The individual settings have been kept constant during the 15 min of aerobic exercise. Mean heart rate for all participants in all activity conditions were in the target range (MeanHR: *M* = 121.52 bpm; *SD* = 8.16). More recent recommendations increasingly advocate the use of subjective exertion as an indicator for relative exercise intensity, suggesting a middle effort of 5–6 on a hypothetical, personal scale from 0 to 10 (Physical Activity Guidelines Advisory Committee Report, 2018) for moderate exercise intensity. Figure 2E indicates moderate intensity by using the latter indicator of a middle effort of subjective exertion (Please note that we used the classic RPE (Borg, 1998) ranging from 6 to 20, i.e., middle effort corresponds to ∼13). Afterward, participants cycled for two more minutes, gradually slowing down, as a cool-down.

Before (T1), immediately after (T2), 10 min after (T3) and 30 min after (T4) the exercise, participants filled out self-report measures on state positive/negative affect (PAAS; Lox et al., 2000), subjective exertion (Rating of Perceived Exertion, RPE; Borg, 1998). During the last minute of the exercise, participants answered the Feeling Scale (FS; Hardy and Rejeski, 1989). This way, the attentional focusing task has not been interrupted by the assessment of state affect, while still being conducted within the timeframe of the exercise. Also, the feeling scale has been performed before the cool-down, ensuring it does capture the affect caused by the exercise and not reflect the reaction to the end of it. At T2, participants additionally indicated their attentional focus during the exercise (see Supplementary Material). Between T2 and T3, participants had a 10-min break, where they were asked to remain seated on a chair. Between T3 and T4, participants completed the experimental His-Mine paradigm measuring emotional evaluation of self-related emotional stimuli (Herbert et al., 2011a,b, 2018; Winter et al., 2015, 2018; Weis and Herbert, 2017; Meixner and Herbert, 2018; Meixner et al., 2019). This paradigm was included in the protocol as an experimental measure to investigate effects of acute aerobic exercise on subsequent affective evaluation of self- and other-related verbal stimuli, as part of a larger study. Analyses regarding data from the His-Mine paradigm differed in objective, research question, analytic methods, and conclusions, focusing on information processing of verbal emotional information. They will therefore be presented and discussed independently from the psychophysiological data in a separate manuscript of the authors. A full overview over the experimental sessions is given in Table 1.

### Materials

Prior to the laboratory sessions, participants completed an online questionnaire assessing, among others, clinically relevant symptoms of depression or anxiety due to their association with negative mood and their interaction with the His-Mine paradigm (Herbert et al., 2011a,b, 2018; Winter et al., 2015, 2018; Weis and Herbert, 2017; Meixner and Herbert, 2018; Meixner et al., 2019). Measures assessed depressive symptomatology (BDI; Hautzinger et al., 2009), dysfunctional attitudes toward oneself (DAS; Hautzinger et al., 2005), state and trait anxiety (STAI; Laux et al., 1981), general physical activity^3^ (GPAQ; Armstrong and Bull, 2006), state and trait affect (PANAS; Watson et al., 1988; Krohne et al., 1996) and habitual attentional focus (Stevinson and Biddle, 1998; Wininger and Gieske, 2010; see Supplementary Material for exact wording). Clinically relevant scores on BDI or STAI as well as high values on the DAS scale, or abnormally high physical activity indicated in the GPAQ would have led to participants not being invited to the laboratory sessions. Habitual attentional focus has been assessed to gain additional insight if participants’ self-reported habitual attentional focus predicts their actual employed attentional focus in the laboratory setting. An overview on participants’ self-report data (age, affect (trait), anxiety, as well as regular, vigorous physical activity) is provided in Table 2.

**TABLE 2:** Descriptive statistics, PANAS, STAI (Trait) and regular physical activity scores (sum scores as suggested by the respective manuals) of the study sample.

Variable	*n*	Mean	Std. Deviation	Reliability (Cronbach’s α )
Age	30	21.00 (2.17)	2.17	–
BMI	30	21.13 (2.27)	2.27	–
Positive affect, trait (PANAS)	30	34.27 (6.13)	6.13	0.88
Negative affect, trait (PANAS)	30	17.33 (4.64)	4.64	0.81
Anxiety, trait (STAI)	30	38.30 (8.82)	8.82	0.90
Vigorous activity/week in minutes (GPAQ)	30	172.00 (225.28)	225.28	–

### Assessment of Impedance Cardiography (ICG) and Data Reduction

Impedance cardiography (ICG) was recorded during the whole experiment using the 7-electrode version of the VU-AMS 5fs device (ambulatory monitoring system; Vrije Universiteit)^4^. Data was cut into multiple intervals, and fixed non-overlapping windows were selected for analysis: Window size for baseline, activity/inactivity and late recovery (i.e., second half of recovery period after activity) was set at 4 min (see Willemsen et al., 1996; Houtveen et al., 2002) from the middle of the interval, i.e., cutting out the first 30s of every interval. For more details on software and filter settings, as well as methods of parameter extraction, please see Supplementary Material.

### Data Analysis

Analyses were performed using IBM SPSS Statistics 24. A manipulation check (3.1) was performed via paired-samples *t*-tests: For each condition, the self-reported amount of time spent with the *instructed* attentional focus was compared to the amount of time participants focused on anything else. Furthermore, to ensure participants’ personality did not notably interact with attentional focus and confound results, Pearson correlation analyses have been performed.

Due to a sample size of *n* = 30 with 4 points of measurement, i.e., 120 measurements in total, warranting assumption of normality and to prevent random results via multiple testing, the general analysis strategy will be as follows: firstly, multiple dependent variables will be grouped together and analyzed using MANOVA, reducing alpha inflation. Subsequently, *post hoc* ANOVAs will be used to further examine significant differences found in the MANOVA results, or dependent variables running counter to each other.

To test the effects of attentional focus on subjective exertion (RPE) and affect (subscales of the PAAS; 3.2), a 4 (*conditions*) × 4 (*time*: *before/after/10 min after/30 min after*) repeated measures MANOVA has been calculated. Feeling scale (FS) has been calculated in a separate 4 (*conditions*) × 4 (*time*: *before/during/after/10 min after/30 min after*) repeated measures ANOVA, since it has been measured at 5 points (additionally during the exercise), and could therefore not be included in the first MANOVA.

Physiological responses (including PEP, CO, SV, RSA, HF, RMSSD, HR, LVET; 3.3) as a function of the different attentional focus conditions were analyzed using a 4 (conditions) × 3 (time: before/during/after activity) repeated measures MANOVA, followed by respective ANOVAs if significant differences were confirmed. To evaluate recovery and parasympathetic reactivation in detail, heart rate recovery (HRR) has been additionally evaluated at 7 points of measurement (Baseline/Warmup/Activity/Cool-down/HRR180/HRR330/HRR480), and RSA and HF at 10 points of measurement (30 s periods from 180 to 480 s after the end of the activity, log-transformed data) using ANOVAs.

Lastly, self-reported focus of attention in conditions C (exercise without attentional focus instruction) and D (inactive control condition, without attentional focus instruction) and self-reported affect, subjective exertion, and psychophysiological responses have been correlated using Person correlation analysis (3.4). This has been done to gain additional insight for future research into possible effects of self-reported attentional focus.

If assumptions of sphericity have been violated, Greenhouse-Geisser correction has been applied. Epsilon values (ϵ) are reported, as well as uncorrected degrees of freedom, and corrected *p*-values. As a measure of effect size, partial eta square (η*_*p*_*^2^) is reported. Multiple correlational analyses (3.4) have been subject to Bonferroni correction.

## Results

### Manipulation Check

Participants indicated following the instructions: During the internal-relevant focus, the internal focus was dominant (all *p* ≤ 0.001). During the external-irrelevant focus condition, the external attentional focus was also dominant, but the differences did not reach statistical significance (all *p* > 0.1). During the exercise condition without attentional focus, participants reported to focus on internal-irrelevant stimuli more often than on any other category (all *p* ≤ 0.001).

Analysis of participants’ habitual attentional focus revealed a preferred focus on internal-irrelevant (e.g., daydreaming, imagining music, etc.) stimuli during physical activity. Participants indicated that during recreational physical activity, they usually focused on average on internal-relevant stimuli *M* = 27.13% (*SD* = 27.87), on external-relevant *M* = 15.43% (*SD* = 20.44), on internal-irrelevant *M* = 38.30% (*SD* = 25.68) and on external-irrelevant stimuli *M* = 21.70% (*SD* = 16.33) of the time. Also, habitual attentional focus was unrelated to personality variables BIS, BAS, FFFS, trait anxiety, dysfunctional attitudes, depressive symptomatology, BMI, minutes of recreational exercise, and general activity in MET minutes (all *p* > 0.1).

Figure 1 depicts participants’ mean responses to the focus-reinforcing questions on whether the force production of their quad muscles (condition A) or the brightness in the track simulation (condition B) has changed.

### Self-Reported Affect and Exertion

A 4 (*conditions: internal-relevant focus, external-irrelevant focus, exercise control condition, inactive control condition*) × 5 (*time*: *T1/during activity/T2/T3/T4*) repeated measures ANOVA (DV: affect as measured by the Feeling Scale) revealed a significant main effect of *time* of the measurement [*F*(4, 460) = 9.51, *p* ≤ 0.001, η*_*p*_*^2^ = 0.11, ϵ = 0.80]. No main effect of *condition* [*F*(3, 115) = 0.38, *p* > 0.1, η*_*p*_*^2^ = 0.01, ϵ = 0.91], nor an interaction *time* × *condition* [*F*(12, 460) = 1.50, *p* > 0.1, η*_*p*_*^2^ = 0.04, ϵ = 0.80] was observed.

A 4 (*conditions*) × 4 (*time*) repeated measures ANOVA (DV: subjective exertion, RPE) revealed a main effect of *time* [*F*(3, 348) = 73.19, *p* ≤ 0.001, η*_*p*_*^2^ = 0.39, ϵ = 0.91], a main effect of *condition* [*F*(3, 116) = 3.11, *p* ≤ 0.05, η*_*p*_*^2^ = 0.08], and an interaction *time* × *condition* [*F*(9, 348) = 7.22, *p* ≤ 0.001, η*_*p*_*^2^ = 0.16, ϵ = 0.91]. *Post hoc* tests revealed significant increases in subjective exertion from pre- to post-activity and a decrease of perceived exertion until the end of the recovery phase, but only in the exercise conditions (all *p* ≤ 0.001).

Regarding PAAS and its subscales (positive affect PA, negative affect NA, fatigue FTG, tranquility TRQ), a 4 (*conditions*) × 4 (*time*) repeated measures MANOVA and subsequent ANOVAs display the same pattern of a persistent main effect of *time* [PA: *F*(3, 345) = 45.61, *p* ≤ 0.001, η*_*p*_*^2^ = 0.28, ϵ = 0.82; NA: *F(*3, 345) = 10.91, *p* ≤ 0.001, η*_*p*_*^2^ = 0.09, ϵ = 0.78; FTG: *F(*3, 345) = 18.76, *p* ≤ 0.001, η*_*p*_*^2^ = 0.14, ϵ = 0.81; TRQ: *F*(3, 345) = 26.57, *p* ≤ 0.001, η*_*p*_*^2^ = 0.19, ϵ = 0.91; but not of *condition*: PA: *F*(3, 115) = 0.28, *p* > 0.1, η*_*p*_*^2^ = 0.01; NA: *F*(3, 115) = 0.18, *p* > 0.1, η*_*p*_*^2^ = 0.01; FTG: *F*(3, 115) = 0.17, *p* > 0.1, η*_*p*_*^2^ = 0.00; TRQ: *F*(3, 115) = 0.42, *p* > 0.1, η*_*p*_*^2^ = 0.01]. Only the subscale positive affect showed an interaction of *time* × *condition* [PA: *F*(9, 345) = 2.79, *p* ≤ 0.01, η*_*p*_*^2^ = 0.07, ϵ = 0.82; NA: *F*(9, 345) = 0.24, *p* > 0.1, η*_*p*_*^2^ = 0.01, ϵ = 0.78; FTG: *F*(9, 345) = 0.34, *p* > 0.1, η*_*p*_*^2^ = 0.01, ϵ = 0.81; TRQ: *F*(9, 345) = 0.87, *p* > 0.1, η*_*p*_*^2^ = 0.02, ϵ = 0.91].

*Post hoc* tests revealed a decrease in negative affect and tranquility, while positive affect, fatigue and tranquility stayed the same, from pre- to post-exercise. Until *10 min after* the exercise, RPE and positive affect decreased, negative affect remained unchanged, and fatigue and tranquility increased. Until the last measurement T4, RPE increased again, positive affect further decreased, negative affect increased again, fatigue further increased, and tranquility decreased again. Regarding condition D, positive affect decreased from *before* to *after* to *10 min after* the inactivity (sitting on the ergometer) and remained stable until *30 min after* (all *p* ≤ 0.05). Results are depicted in Figures 2A–F.

### Physiological Responses to Different Attentional Focus Conditions

It has been hypothesized that attentional focus instructions might influence physiological activity elicited during aerobic exercise due to additional demands placed upon the active person.

#### Physiological Responses (PEP, CO, SV, RSA, HF, RMSSD, HR, LVET)

**FIGURE 3:**
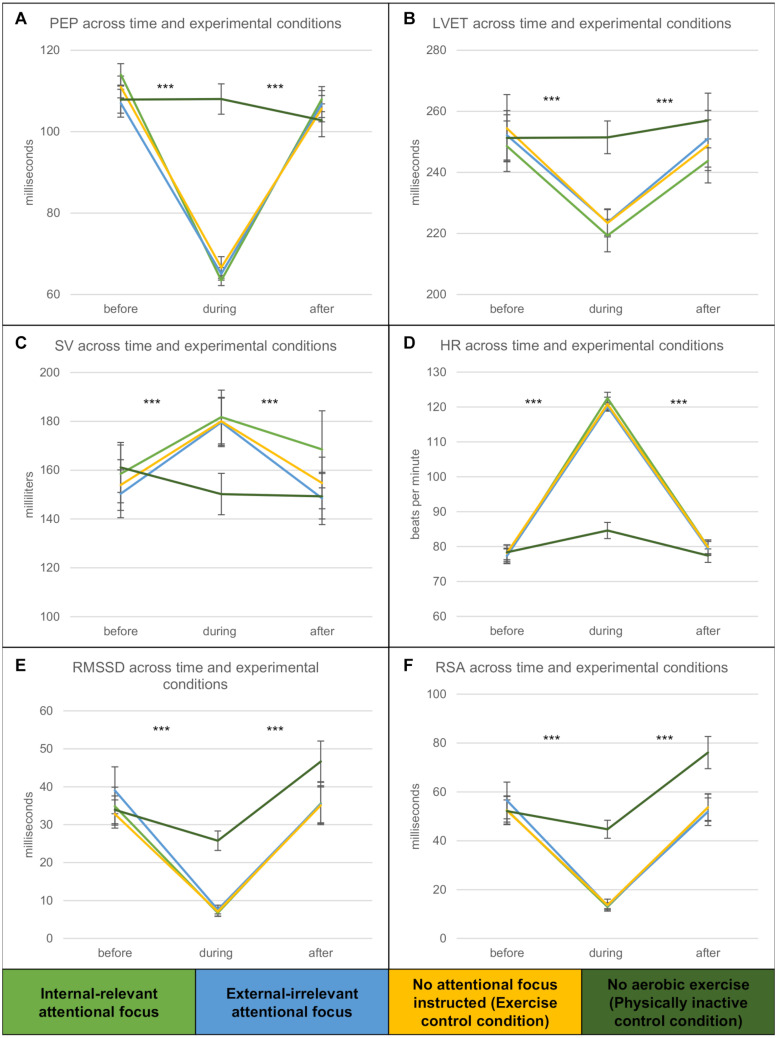
**(A–F)** Changes in PEP (ms), LVET (ms), SV (cm^3^/ml), HR (bpm), RMSSD (ms), and RSA (ms) across time (before, during and + 10min after the (in-) activity) and experimental conditions. Asterisks indicate significant differences between time points, regardless of exercise condition (*p < 0.05; **p < 0.01; ***p < 0.001). Condition D (physically inactive) is depicted for additional reference (Note: Since physical inactivity in condition D has been shorter than the exercise in ABC, data from a later point in time is depicted for condition D to keep times congruent). Vertical bars denote +/− standard errors.

Descriptively, physiological parameters during baseline and aerobic exercise were in the expected physiological ranges (Goedhart et al., 2006; Willemsen et al., 1996), e.g., HR during rest (sitting, baseline) *M* = 77.81 bpm, *SD* = 11.79; HR during rest (sitting on bicycle) *M* = 84.62 bpm, *SD* = 12.73; HR during cycling at moderate intensity *M* = 121.17 bpm, *SD* = 8.95; PEP during rest (sitting, baseline) *M* = 109.96 ms, *SD* = 16.66; PEP during rest (sitting on bicycle) *M* = 108.00 ms, *SD* = 20.40; PEP during aerobic exercise *M* = 65.04 ms, *SD* = 10.44; SV during rest (sitting, baseline) *M* = 155.96 cm^3^, *SD* = 57.36; SV during rest (sitting on bicycle) *M* = 150.22 cm^3^, *SD* = 46.64; SV during aerobic exercise *M* = 180.44 cm^3^, *SD* = 55.58. Figures 3A–F depict physiological changes across time and experimental conditions.

Using Pillai’s trace, there was not a significant effect of *condition* on any physiological parameter [PEP, CO, SV, RSA, HF, RMSSD, HR, LVET; *V* = 0.14, *F*(16, 142) = 0.69, *p* > 0.1, η*_*p*_*^2^ = 0.07] but a significant effect of *time* [*V* = 0.97, *F*(16, 62) = 108.41, *p* ≤ 0.001, η*_*p*_*^2^ = 0.97] and no interaction *time* × *condition* [*V* = 0.31, *F*(32, 126) = 0.72, *p* = 0.86, η*_*p*_*^2^ = 0.16]. Subsequent ANOVAs confirm this pattern for each of the dependent variables (*time*: all *p* ≤ 0.001; *condition*: all *p* > 0.1; *time* × *condition*: *p* > 0.1; for detailed results, please see Supplementary Material).

*Post hoc* tests revealed that PEP, RSA and LVET were significantly reduced during aerobic exercise and returned to baseline levels in the recovery period (all *p* ≤ 0.001), as is expected for aerobic exercise. HR, SV, and CO increased during aerobic exercise to meet physiological demands and returned to baseline level after cessation of the activity (all *p* ≤ 0.001). As it is already evident in the MANOVA results, these patterns did not differ between nor interacted with the experimental exercise conditions. The physically inactive condition D, however, differed from the exercise conditions, such that the analyzed parameters did not notably change during the session (see Figure 3).

#### In-Depth Analysis of Recovery and Parasympathetic Reactivation (RSA, RMSSD, HRR)

To evaluate RSA during the recovery period, and therefore parasympathetic reactivation after the activity, in a more detailed fashion, an additional 3 (*exercise conditions*) × 11 (*time*: 30 s periods from 180 to 480 s after the end of the activity) rmANOVA has been conducted, revealing a main effect of *time* [*F*(10, 860) = 4.28, *p* ≤ 0.001, η*_*p*_*^2^ = 0.05, ϵ = 0.61], but not of *condition* [*F*(2, 86) = 0.04, *p* > 0.1, η*_*p*_*^2^ = 0.00], nor an interaction *time* × *condition* [*F*(20, 860) = 0.90, *p* > 0.1, η*_*p*_*^2^ = 0.02, ϵ = 0.61].

To further evaluate HRV using a time-based parameter (RMSSD), a 3 (*exercise conditions*) × 11 (*time*: 30 s periods from 180 to 480 s after the end of the activity, log-transformed data^5^) rmANOVA demonstrated a significant main effect of *time* [*F*(10, 870) = 6.09, *p* ≤ 0.001, η*_*p*_*^2^ = 0.07, ϵ = 0.71], but not of *condition* [*F*(2, 87) = 0.02, *p* > 0.1, η*_*p*_*^2^ = 0.00], and no interaction of *time* × *condition* [*F*(20, 870) = 0.98, *p* > 0.1, η*_*p*_*^2^ = 0.02, ϵ = 0.71].

Lastly, a 3 (*exercise conditions*) × 7 (*time*: Baseline/Warmup/Activity/Cooldown/HRR180/HRR330/HRR480) repeated measures ANOVA (DV: mean heart rate) revealed a significant main effect of *time* [*F*(6, 522) = 579.59, *p* ≤ 0.001, η*_*p*_*^2^ = 0.87, ϵ = 0.60], but neither a main effect of *condition* [*F*(2, 87) = 0.10, *p* > 0.1, η*_*p*_*^2^ = 0.00], nor an interaction of *time* × *condition* [*F*(12, 522) = 0.36, *p* > 0.1, η*_*p*_*^2^ = 0.01, ϵ = 0.60]. *Post hoc* tests also confirmed that there is no difference between any of the conditions in heart rate, nor heart rate recovery after the exercise (i.e., returning to baseline levels; all *p* > 0.1).

### Correlational Analyses Using Self-Reported Attentional Focus

Pearson correlation analyses have been performed to discover a possible relationship between self-reported attentional focus and affect (FS, PAAS) and perceived exertion (RPE) in conditions C (exercise without attentional focus instruction) and D (no exercise, no attentional focus instruction). For example, a stronger focus on external-relevant stimuli has been associated with more subjective exertion after the exercise, in condition C. In condition D, after the inactivity, a stronger attentional focus on internal-relevant stimuli has been associated with more subjective exertion, whereas a focus on internal-irrelevant stimuli has been associated with less subjective exertion. Table 3 depicts full results on Pearson correlation coefficients for the relationships between self-reported attentional focus and affect (FS, PAAS) and perceived exertion (RPE) in experimental conditions C and D.

**TABLE 3:** Pearson correlation coefficients for self-reported focus of attention and subjective exertion (RPE), positive affect (PAAS), negative affect (PAAS), and positive affect (Feeling Scale).

		Pre	Post	+10 min	+30 min	Pre	Post	+10 min	+30 min	Pre	Post	+10 min	+30 min	Pre	Peri	Post	+10 min	+30 min
					
		Subjective exertion (RPE)				Positive affect (PAAS)				Negative affect (PAAS)				Positive affect (FS)				
Condition C	Internal relevant	0.40*	0.11	0.14	0.26	−0.10	−0.08	0.07	0.12	0.10	0.08	0.12	−0.06	−0.40*	−0.15	−0.28	−0.12	0.01
	External relevant	0.26	0.40*	0.48**	0.33	−0.15	−0.09	−0.04	−0.03	−0.06	0.13	0.16	−0.14	−0.36	−0.14	−0.42*	−0.29	0.04
	Internal irrelevant	−0.16	−0.24	−0.27	0.02	−0.06	−0.21	−0.29	−0.36	0.09	0.13	0.08	0.22	0.10	0.16	0.09	−0.13	−0.21
	External irrelevant	0.00	0.23	0.13	−0.18	−0.05	0.09	0.06	0.15	−0.07	−0.22	−0.19	−0.17	0.11	0.23	0.14	0.32	0.20
Condition D	Internal relevant	0.12	0.45*	0.40*	0.06	−0.05	0.18	0.00	−0.03	−0.10	0.13	0.01	−0.23	0.02	0.08	0.06	0.28	0.26
	External relevant	0.22	0.31	0.01	0.33	−0.28	−0.18	−0.20	−0.24	0.12	0.06	0.08	0.00	−0.30	0.14	0.16	0.02	−0.14
	Internal irrelevant	−0.22	−0.57**	−0.41*	−0.16	0.16	−0.07	0.12	0.15	0.10	−0.06	0.02	0.31	0.18	−0.14	−0.02	−0.18	−0.17
	External irrelevant	0.20	0.35	0.25	0.06	−0.15	−0.02	−0.11	−0.08	−0.10	−0.09	−0.08	−0.23	−0.32	0.00	−0.24	−0.12	−0.01

Calculated separately for condition C (exercise without attentional focus instruction) and condition D (control condition without exercise and attentional focus instruction). Please note that p-values are reported uncorrected and none of the significant correlations would survive Bonferroni correction. *p < 0.05, **p < 0.01, ***p < 0.001.

Lastly, Pearson correlation analyses have been performed to examine the relationship between the self-reported time spent with an internal-irrelevant, internal-relevant, external-irrelevant, external-relevant focus and physiological responses at baseline (pre), during the activity (peri) and 10 min after the activity (post). For example, in condition C, a stronger focus on internal-irrelevant stimuli has been associated with a shorter PEP (i.e., less milliseconds), pre-, peri-, and post-exercise; and with a higher average HR peri- and post-exercise. In condition D, a stronger focus on internal-irrelevant stimuli was associated with higher RMSSD before and after the period of inactivity. Table 4 depicts Pearson correlation coefficients for the relationships between the self-reported time spent with an internal-irrelevant, internal-relevant, external-irrelevant, external-relevant focus and physiological responses. However, please note that *p*-values are reported uncorrected and none of the significant correlations would survive Bonferroni correction.

**TABLE 4:** Pearson correlation coefficients for self-reported focus of attention and pre-ejection period (PEP), average HR, heart rate variability (RMSSD and HF), left-ventricular ejection time (LVET), stroke volume (SV), cardiac output (CO), and respiratory sinus arrythmia (RSA).

		Pre	Peri	Post	Pre	Peri	Post	Pre	Peri	Post	Pre	Peri	Post	Pre	Peri	Post	Pre	Peri	Post	Pre	Peri	Post	Pre	Peri	Post
									
		PEP			Average HR			RMSSD			HF			LVET			SV			CO			RSA		
Condition C	Internal relevant	0.20	0.21	0.27	−0.13	−0.14	−0.18	−0.06	−0.15	0.06	−0.08	−0.20	0.08	0.27	0.17	0.12	−0.05	−0.17	−0.15	−0.12	−0.21	−0.20	−0.14	−0.17	−0.02
	External relevant	0.22	0.00	0.32	−0.09	−0.07	−0.09	−0.08	−0.07	−0.04	−0.15	−0.11	−0.07	0.29	0.35	0.23	0.05	−0.15	−0.07	−0.02	−0.16	−0.09	−0.15	−0.12	−0.08
	Internal irrelevant	−0.38*	−0.19	−0.53**	0.26	0.40*	0.37*	−0.12	−0.27	−0.19	−0.11	−0.24	−0.15	−0.12	−0.29	−0.03	0.01	0.04	0.06	0.15	0.15	0.21	−0.13	−0.28	−0.19
	External irrelevant	0.22	0.05	0.32	−0.11	−0.29	−0.22	0.08	0.08	0.15	0.10	−0.20	0.10	−0.37*	0.03	−0.31	−0.32	−0.10	−0.21	−0.33	−0.18	−0.29	0.04	0.08	0.06
Condition D	Internal relevant	0.09	−0.07	−0.51	0.15	0.09	0.37*	−0.35	−0.27	−0.42*	−0.25	−0.25	−0.36	0.23	0.12	0.08	0.22	0.13	0.67	0.29	0.16	0.82*	−0.36	−0.25	−0.36
	External relevant	0.06	0.02	0.21	0.07	0.13	−0.09	−0.11	−0.12	−0.13	−0.14	−0.12	−0.13	0.01	−0.07	0.73	0.08	0.07	0.11	0.12	0.10	−0.06	−0.11	−0.07	−0.16
	Internal irrelevant	−0.09	0.22	0.25	−0.27	−0.31	−0.26	0.45*	0.45*	0.51**	0.33	0.41*	0.45*	−0.11	0.03	−0.88*	−0.31	−0.28	−0.66	−0.43*	−0.38*	−0.49	0.38*	0.41*	0.40*
	External irrelevant	−0.06	−0.45*	0.08	0.28	0.51**	0.13	−0.20	−0.34	−0.28	−0.10	−0.31	−0.25	−0.15	−0.24	0.82*	0.30	0.37*	0.17	0.43*	0.55**	0.20	−0.03	−0.28	−0.12

Calculated separately for condition C (exercise without attentional focus instruction) and condition D (control condition without exercise and attentional focus instruction). Please note that p-values are reported uncorrected and none of the significant correlations would survive Bonferroni correction. *p < 0.05, **p < 0.01, ***p < 0.001.

## Discussion

We investigated the affective and physiological effects of attentional focus during an acute bout of moderate cycling exercise. It was hypothesized that the attentional focusing might resemble an additional task, increasing the total psychophysiological demands of the activity and therefore possibly influencing affective and physiological responses. To test this hypothesis, attentional focus was experimentally manipulated to be directed at internal-relevant or external-irrelevant stimuli during a cycling task. The presented results question the importance of participants’ attentional focus during exercise for affect, subjective exertion and physiological responses and recovery.

In general, the attentional focus manipulation can be considered successful: experimentally induced focus has also been reported to be dominant in the respective conditions. Furthermore, it is also evident that the track simulation gave ample brightness changes to focus on, and that participants displayed a natural cycling performance, with a non-linear flow of force production. It is also reasonable to assume that most participants were engaged in the task until the end, given the displayed variance in answers (Figure 1): Since it could be expected that mentally absent (e.g., focusing on internal-irrelevant stimuli, i.e., daydreaming) participants would not have noticed any changes in force production or brightness, the answer “constant” would have occurred much more frequently than data suggests.

Although the attentional focus manipulation can be considered successful, neither participants’ affective reaction toward the cycling exercise, nor their perceived exertion, nor any physiological parameter differed by experimental condition, apart from the inactive control condition. This is not in line with previous studies reporting an additive effect of combined mental and physical challenges (Roth et al., 1990; Taelman et al., 2011), but rather supportive of contrary research (e.g., Wasmund et al., 2002; Greig et al., 2007). Therefore, it seems reasonable to assume that attentional focusing cannot be considered an adequate challenge to sufficiently influence the cardiovascular reactions to exercise. Although the task of attentional focusing has been designed as an active task with reinforcement—i.e., being regularly reminded and asked to state out loud if the quality of the ambient light or the sensation of their quad muscles has changed—it is obviously not comparable to challenges such as mental arithmetic used by previous research.

With respect to physiological responses to cycling exercise, it is evident that the additional task of instructing an attentional focus did not further modulate sympathetic activation during or parasympathetic reactivation after exercise compared to exercise without attentional focus instructions. At the same time, previous literature suggesting a masked, or blunted psychophysiological response due to an additional mental task (Greig et al., 2007) could also not be supported. Not even a deliberately chosen attentional focus on internal-irrelevant stimuli seems to exert influence on the psychophysiological reactions to exercise. However, this was the case in short bouts of acute, moderate cycling exercise and can’t be generalized toward higher intensities, prolonged durations, other exercise types (e.g., running) nor toward long-term effects of combined mental and physical challenges.

Another limitation of our study is the restricted sample of active females. Firstly, the results can therefore not be generalized toward a male sample. As outlined in the introduction, including male participants would have led to numerous interactions. Future studies, however, could take a step further and double the sample size to compare male to female participants in this experimental set-up. Secondly, the results cannot be generalized toward sedentary samples or high-performance athletes, with both groups possibly having a notably different approach to and knowledge about attentional focus during exercise.

Two further implications of the presented results should be discussed: Firstly, our study did not find any meaningful differences between all exercise conditions (cycling with an internal vs. external vs. no attentional focus) and could therefore not replicate previous findings. Contrary to previous studies assessing the thoughts of participants during exercise (Lind et al., 2009; Brick et al., 2014) we experimentally manipulated attentional focus: The beneficial effects of an external focus of attention might be tied to voluntariness or flexibility in the choice of attentional focus within the “external-irrelevant” category. Alternatively, the hypothesized benefits of an external-irrelevant focus of attention, or distraction, i.e., reduction in tension, increase in revitalization and positive engagement, might be most present in outdoor environments (Thompson Coon et al., 2011).

Secondly, if cardiovascular activity remains unchanged by an instructed attentional focus, this imposes the question as to what is responsible for the often-reported negative affect and increased subjective exertion after exercise with an internal focus of attention. If cardiovascular reactions are comparable, the instructed attentional focus possibly influences either sensitivity to, or appraisal processes of the physical symptoms during exercise, leading to an altered affective experience.

Although the proposed hypotheses could not be supported by the data, additional analyses, i.e., of participants’ self-chosen attentional focus during exercise without attentional focus instructions, provided some insight for further research: If not specifically instructed, participants mostly chose internal-irrelevant stimuli to focus on during the exercise in a laboratory setting, suggesting this to be the most attractive distraction in this particular setting. Also, looking at the correlational analyses (Tables 3, 4), internal-irrelevant focus could be associated with subjective exertion and psychophysiological responses to activity and inactivity. Although these correlations did not survive Bonferroni correction for multiple testing, they might inspire future hypotheses and experiments on the subject. Therefore, we recommend including a variety of experimentally manipulated attentional focus, e.g., on internal-irrelevant stimuli such as imagining music, philosophy, creating mental to-do-lists, or mental arithmetic, for future research, as well as assessment of self-reported attentional focus. Additional performance-based or physiological parameters (e.g., pacing, oxygen consumption; Schücker et al., 2009, 2016a,b) could also aid in entangling the effects of attention during physical activity even further. From these results, no specific recommendations can be formulated for non-professional exercisers. However, for future experimental research in sports psychology or sports physiology, these results imply that internal-relevant and external-irrelevant attentional focus are low-impact manipulations, placing no added mental demands on top of the physical demands by the exercise. Besides that, the results of this study also strongly suggest that attentional focus during exercise might generally be not as important to affect, subjective exertion or cardiovascular activity as has been suggested in the past.

## Data Availability Statement

The datasets presented in this article are not readily available because due to the informed consent form in which the possibility of raw data being published online was not explicitly stated, only group-level data, as it is provided in this manuscript, will be made accessible. All authors contributed to the article and approved the submitted version.

## Ethics Statement

The studies involving human participants were reviewed and approved by the Ethics Committee of Ulm University (https://www.uni-ulm.de/einrichtungen/ethikkommission-der-universitaet-ulm/). The patients/participants provided their written informed consent to participate in this study.

## Author Contributions

FM prepared the manuscript, tables, and figures. CH revised it for scientific and intellectual content. FM planned the experiment, collected the data, preprocessed and analyzed the results under the supervision of CH. Both authors contributed to the article and approved the final version of the manuscript.

## Conflict of Interest

The authors declare that the research was conducted in the absence of any commercial or financial relationships that could be construed as a potential conflict of interest.

## Footnotes

1 http://www.ergoplanet.de/

2 According to the American Heart Association 50–70% of HRMax corresponds to moderate intensity (American Heart Organization: Target Heart Rates Chart, 2015). However, to account for other recommendations with a higher range, e.g., by the U.S. Department of Health and Human Services (Physical Activity Guidelines Advisory Committee Report, 2008); 64–76% of HRMax), but to avoid the ventilatory threshold, a range from 60 to 70% of HRMax has been chosen.

3 Participants did not regularly exercise on a bicycle ergometer but did use a bicycle multiple times per week.

4 www.vu-ams.nl

5 Especially when using Ultra-Short Term HRV measurements, it is recommended to log-transform the data before applying parametric methods (e.g., Esco and Flatt, 2014; Pecchia et al., 2018). Using raw data does not alter the presented results.
