# Supplementary material for: Does Attentional Focus Influence Psychophysiological Responses to an Acute Bout of Exercise? Evidence From an Experimental Study Using a Repeated-Measures Design
Source: Front Physiol. 2021 Jun 25;12:680149. doi: 10.3389/fphys.2021.680149 (PMC8267581; doi:10.3389/fphys.2021.680149)
Supplement: Supplementary file 1 [file Data_Sheet_1.pdf]

Supplementary Material

SUPPLEMENT 1

VU-DAMS Configuration

Physiological data (ECG and ICG-dZ) was sampled at 1000 Hz, ICG-Z0 was sampled at 250 Hz. Using the VU-DAMS software (Vrije Universiteit; www.vu-ams.nl), ECG data was inspected for artifacts and semi-automatically artifact-corrected, i.e. each automatically detected artifact was manually checked by the experimenter. ECG artifacts (M=0.31 %, SD=0.014 throughout the recordings) were discarded and missing data extrapolated using cubic spline interpolation whenever <5 consecutive beats have been discarded. Artifact detection and rejection followed the VU-AMS guidelines and default settings implemented in the VU-DAMS software. ICG data has been semi-automatically artifact-corrected using the VU-DAMS default impedance scoring algorithm, and manually checked by the experimenter afterwards. ICG artifacts (M=4.65 %, SD=3.96 throughout the recordings) were subsequently discarded.

Heart Rate (HR), inter-beat intervals (IBI), and subsequent HRV parameters (HF, RMSSD) were extracted from the R-wave peaks in the continuous ECG signal. PEP was measured as the period from the Q-Point in the QRS complex of a heartbeat (ECG) to the B-Point (opening of the aortic valves) in the thoracic impedance changes over time DZ/DT (ICG) in milliseconds. LVET has been measured as the time from the B-point to the X-point (closure of the aortic valve) in the DZ/DT signal in milliseconds. SV has been calculated as the amount of blood ejected per beat in cm^3 using the distance between the frontal ICG electrodes and the thoracic impedance changes. CO (or minute volume) has been calculated using the formula (SV*HR*0.001). RSA was extracted using the peak-valley method (Grossman et al., 1990; de Geus et al., 1995; Willemsen et al., 1996), using the ECG signal, IBIs, and the respiration signal, calculated from thoracic impedance.

Data was cut into multiple intervals: Interval length for baseline, activity/inactivity and late recovery (i.e. second half of recovery period after activity) was set at 4 minutes from the middle of the interval (Willemsen et al., 1996; Houtveen et al., 2002) For each time period, heart rate (HR), (ultra-) short-term heart rate variability (HRV; parameters HF, RMSSD), pre-ejection period (PEP), left-ventricular ejection time (LVET), stroke volume (SV), cardiac output (CO), and respiratory sinus arrythmia (RSA), have been calculated using ECG signals and thoracic impedance (ICG) and subsequently averaged.

Initial recovery from physical activities always contains considerable drops in heart rate and overall sympathetic activation, making it impractical to evaluate post-exercise recovery as an undivided period. To gain a more reliable measure of recovery effects, this period was split into smaller 30-seconds periods, following the recommendations of Goldberger (Goldberger et al., 2006) regarding the assessment of post-exercise HRV or parasympathetic reactivation (results in Supplement, section “In-depth analysis of recovery and parasympathetic reactivation”.

SUPPLEMENT 2

Assessment of habitual and situational attentional focus (based on (Wininger and Gieske (2010), albeit with categories based on Stevinson and Biddle (1998)). Participants were asked to indicate their responses on a visual analogue scale (0-100% of the time).

**Attentional Focus** *(not visible to participants)*	**Instruction**: Please indicate if / how much of the time you [spend during a typical physical activity] *or* [spent during the 15 minutes of physical activity] with the following categories.
Internal-relevant	Your attention is directed inwards, i.e. how your body feels during the exercise – Examples are breathing, muscle soreness, thirst, fatigue, sweating, blisters, or nausea.
External-relevant	Your attention is directed outwards on things that are relevant for the execution of the task – Examples are strategy, track markers, water stations, lap times, the track and its conditions.
Internal-irrelevant	Your attention is directed inwards on things that are irrelevant for the execution of the task – Examples are daydreaming, imagining music, arithmetic problems, philosophy, or religion.
External-irrelevant	Your attention is directed outwards on things that are irrelevant for the execution of the task – Examples are scenery, spectators, other exercising people, or the general environment.

SUPPLEMENT 3

Training/Repetition Effects

A MANOVA (comparing sessions from weeks 1-4, regardless of experimental condition) has been performed to check for training / repetition effects and for the stability of physiological parameters at baseline acquired during this experiment. Using Pillai's trace, there was not a significant effect of the point of measurement on any physiological parameters (resting HR, HF, RMSSD, PEP, LVET CO, SV, RSA) at baseline measurements, V=0.25, $F(24, 240)=0.92$, $p=0.58$, $\eta_p^2=0.08$.

SUPPLEMENT 4

Detailed results on psychophysiological data (MANOVA, section 3.3)

DV	time	condition	time x condition
PEP	($F(2, 154)=385.48$, $p\leq0.001$, $\eta_p^2=0.83$, $\epsilon=0.73$)	($F(2, 77)=0.78$, $p>0.1$, $\eta_p^2=.02$)	($F(4, 154)=0.97$, $p>0.1$, $\eta_p^2=0.03$, $\epsilon=0.73$)
CO	($F(2, 154)=213.20$, $p\leq0.001$, $\eta_p^2=0.73$, $\epsilon=0.82$)	($F(2, 77)=0.46$, $p>0.1$, $\eta_p^2=.01$)	($F(4, 154)=0.46$, $p>0.1$, $\eta_p^2=0.01$, $\epsilon=0.82$)
SV	($F(2, 154)=12.85$, $p\leq0.001$, $\eta_p^2=0.14$, $\epsilon=0.96$)	($F(2, 77)=0.73$, $p>0.1$, $\eta_p^2=.02$)	($F(4, 154)=1.00$, $p>0.1$, $\eta_p^2=0.03$, $\epsilon=0.96$)
RSA	($F(2, 154)=121.05$, $p\leq0.001$, $\eta_p^2=0.61$, $\epsilon=0.74$)	($F(2, 77)=0.07$, $p>0.1$, $\eta_p^2=.00$)	($F(4, 154)=0.26$, $p>0.1$, $\eta_p^2=0.01$, $\epsilon=0.74$)
HF	($F(2, 154)=22.70$, $p\leq0.001$, $\eta_p^2=0.23$, $\epsilon=0.74$)	($F(2, 77)=0.13$, $p>0.1$, $\eta_p^2=.00$)	($F(4, 154)=0.47$, $p>0.1$, $\eta_p^2=0.01$, $\epsilon=0.74$)
RMSSD	($F(2, 154)=94.50$, $p\leq0.001$, $\eta_p^2=0.55$, $\epsilon=0.72$)	($F(2, 77)=0.17$, $p>0.1$, $\eta_p^2=.01$)	($F(4, 154)=0.41$, $p>0.1$, $\eta_p^2=0.01$, $\epsilon=0.72$)
HR	($F(2, 154)=857.84$, $p\leq0.001$, $\eta_p^2=0.92$, $\epsilon=0.65$)	($F(2, 77)=0.09$, $p>0.1$, $\eta_p^2=.00$)	($F(4, 154)=0.01$, $p>0.1$, $\eta_p^2=0.00$, $\epsilon=0.65$)
LVET	($F(2, 154)=18.07$, $p\leq0.001$, $\eta_p^2=0.19$, $\epsilon=0.93$)	($F(2, 77)=0.19$, $p>0.1$, $\eta_p^2=.01$)	($F(4, 154)=0.22$, $p>0.1$, $\eta_p^2=0.01$, $\epsilon=0.93$)
